# Genome of a Gut Strain of *Bacillus subtilis*

**DOI:** 10.1128/genomeA.00184-12

**Published:** 2013-02-14

**Authors:** Ghislain Schyns, Cláudia R. Serra, Thomas Lapointe, José B. Pereira-Leal, Sébastien Potot, Patrick Fickers, John B. Perkins, Markus Wyss, Adriano O. Henriques

**Affiliations:** a Research and Development, DSM Nutritional Products Ltd., Kaiseraugst, Switzerland; b Instituto de Tecnologia Química e Biológica, Universidade Nova de Lisboa, Oeiras, Portugal; c Instituto Gulbenkian de Ciência, Oeiras, Portugal; d Biotechnology and Bioprocess, Université Libre de Bruxelles, Brussels, Belgium

## Abstract

*Bacillus subtilis* is a Gram-positive, rod-shaped, spore-forming bacterium. We present the genome sequence of an undomesticated strain, BSP1, isolated from poultry. The sequence of the BSP1 genome supports the view that *B. subtilis* has a biphasic lifestyle, cycling between the soil and the animal gastrointestinal tract, and it provides molecular-level insight into the adaptation of *B. subtilis* to life under laboratory conditions.

## GENOME ANNOUNCEMENT

There is mounting evidence that *Bacillus subtilis* is not strictly a soil organism, but is instead an organism with a biphasic (soil and gut) life cycle (1–4); there is also evidence regarding the ability of *B. subtilis* spores to germinate in animal gut (5–7). In the animal gut, adaptive evolution has resulted in the establishment of commensal bacteria that live in symbiosis with their host (8). As a consequence of this relationship, the host benefits from the intestinal microflora, which itself impacts host physiology, biology, and immunology (9). The genome sequence we present here is that of an undomesticated strain, formerly isolate 200, renamed BSP1, isolated from organically reared broilers (1), and it must be compared to the genome of *B. subtilis* 168, which was one of the first prokaryotic genomes to be sequenced (10). Recent publications have reinforced the impact of this initial sequence (2, 11–13). BSP1, 168, and Bsn5 are strains of *Bacillus subtilis* subsp. *subtilis* (14; C. R. Serra, A. M. Earl, T. M. Barbosa, R. Kolter, and A. O. Henriques, unpublished data), whereas gtP20b and W23 are *Bacillus subtilis* subsp. *spizizenii* strains (15, 16).

Over 100 Mb (105,914,666 bases through 1,025,436 reads) of raw sequence, translating into an average coverage of 26-fold, was used to assemble the genome of the undomesticated gut-associated *B. subtilis* strain BSP1. The genome consists of a single circular chromosome of 4,043,754 bp with an average G+C content of 43.87%.

Most of the BSP1 coding sequences are conserved and are arranged in a collinear manner in closely related *B. subtilis* 168. However, the circular chromosome in BSP1 is somewhat smaller than that in the closely related *B. subtilis* strain, due mainly to the absence of prophage islands, which are abundant in the *B. subtilis* 168 genome. Deletions covering about 250 kb were detected in the regions equivalent to *B. subtilis* prophages 2 to 7, SPβC2, and the skin element. These excisions have caused the loss of several negative regulators of Spo0A (*rapE, rapK, rapI*, and their companion *phr* genes), a key regulatory protein in many responses to the entry into stationary phase, including sporulation. This explains the important biological properties of BSP1, including its potent antimicrobial activity (AbrB-repressed *sbo-alb* operon; A7A1_0154 to A7A1_0162) and its ability to sporulate during growth, both of which may be beneficial in the gut (Serra et al., unpublished data). BSP1 also carries at least 200 genes not found in *B. subtilis* 168, most of which were presumably acquired horizontally, mainly from the closely related *Bacillus licheniformis*. Some of these genes appear to control complex properties and behaviors, such as colony morphology and biofilm architecture (*bsmA* homolog; A7A1_2008), mucosal adhesion through a sortase-dependent collagen-binding protein (functional YwpE sortase associated with the homologs of Cna-type collagen-binding proteins; A7A1_1084, A7A1_1083, and A7A1_1082), and galactoglucomannan utilization (*gam* genes; A7A1_2761 to A7A1_2769). These genes may represent the signatures of gut strains of *B. subtilis*. Overall, while supporting the view that *B. subtilis* has a biphasic lifestyle, cycling between soil and the animal gastrointestinal tract, the genome sequence also evidences molecular details of the domestication process.

### Nucleotide sequence accession number.

The genome sequence of *B. subtilis* subsp. *subtilis* strain BSP1 was deposited in NCBI GenBank under the accession no. CP003695.
